# CAFs-TAECs空间距离预测肺鳞状细胞癌新辅助化免病理缓解

**DOI:** 10.3779/j.issn.1009-3419.2025.102.30

**Published:** 2025-08-20

**Authors:** Duming YE, Liying YANG, Yimin ZHAO, Yinhui WEN, Miaoqing ZHAO, Ligang XING, Xiaorong SUN

**Affiliations:** ^1^261053 潍坊，山东第二医科大学医学影像学院（叶笃明，孙晓蓉）; ^1^School of Medical Imaging, Shandong Second Medical University, Weifang 261053, China; ^2^250117 济南，山东省肿瘤防治研究院，山东省肿瘤医院核医学科（叶笃明，赵亦民，孙晓蓉）; ^2^Department of Nuclear Medicine, Shandong Cancer Hospital and Institute, Jinan 250117, China; ^3^250117 济南，山东省肿瘤防治研究院，山东省肿瘤医院放疗科（杨丽颖，文印会，邢力刚）; ^3^Department of Radiotherapy, Shandong Cancer Hospital and Institute, Jinan 250117, China; ^4^250117 济南，山东第一医科大学（山东省医学科学院）研究生院（赵亦民）; ^4^Department of Graduate, Shandong First Medical University (Shandong Academy of Medical Sciences), Jinan 250117, China; ^5^646000 泸州，西南医科大学附属医院肿瘤科（文印会）; ^5^Department of Oncology, Affiliated Hospital of Southwest Medical University, Southwest Medical University, Luzhou 646000, China; ^6^250117 济南，山东省肿瘤防治研究院，山东省肿瘤医院病理科（赵苗青）; ^6^Department of Pathology, Shandong Cancer Hospital and Institute, Jinan 250117, China

**Keywords:** 肺肿瘤, 新辅助治疗, 癌症相关成纤维细胞, 肿瘤相关血管内皮细胞, Lung neoplasms, Neoadjuvant therapy, Cancer-associated fibroblasts, Tumor-associated endothelial cells

## Abstract

**背景与目的:**

新辅助治疗策略在非小细胞肺癌（non-small cell lung cancer, NSCLC）的综合治疗中具有重要地位。然而，肺鳞状细胞癌对新辅助治疗疗效普遍优于肺腺癌。本文旨在明确基线癌症相关成纤维细胞（cancer-associated fibroblasts, CAFs）与肿瘤相关血管内皮细胞（tumor-associated endothelial cells, TAECs）对肺鳞状细胞癌和肺腺癌不同新辅助治疗疗效的影响。

**方法:**

回顾性收集2018年1月1日至2023年12月31日在山东省肿瘤医院接受新辅助化疗（neoadjuvant chemotherapy, NAC）或新辅助化免（neoadjuvant chemoimmunotherapy, NAIC）治疗的104例II-III期NSCLC患者的治疗前活检样本。全自动组织微阵列制作仪构建组织芯片，并运用多色免疫荧光（α-SMA/CD31/CK/DAPI）技术染色CAFs（α-SMA^+^/CK^-^）和TAECs（CD31^+^/CK^-^），定量CAFs和TAECs的密度、CAFs与TAECs的最近邻距离及接近度（30 μm）。χ^2^检验确定组间主要病理缓解（major pathological response, MPR）差异，其定义为经新辅助治疗后，术后切除标本中存活肿瘤细胞比例≤10%。*Mann-Whitney U*检验分析定量资料组间差异，受试者工作特征（receiver operating characteristic, ROC）曲线确定免疫指标对NAIC MPR的预测性能。

**结果:**

104例接受新辅助治疗的NSCLC患者中，35例接受了NAIC，69例接受了NAC。在总体患者中，肺鳞状细胞癌较肺腺癌更易发生MPR（50.0% *vs* 22.4%, *P*=0.006）；在NAIC患者中，这一趋势仍然存在（72.7% *vs* 30.8%, *P*=0.038）；在NAC患者中，两者的MPR率无差异。NAIC或NAC治疗前，肺鳞状细胞癌与肺腺癌的程序性死亡配体1（programmed death ligand 1, PD-L1）/程序性细胞死亡受体1（programmed cell death 1, PD-1）表达、CAFs及TAECs的密度、CAFs与TAECs的最近邻距离、CAFs与TAECs的接近度（30 μm）均无差异。在接受NAIC治疗的肺鳞状细胞癌患者中，MPR较NMPR基线瘤内PD-L1/PD-1表达、CAFs及TAECs的密度无统计学差意义，而CAFs与TAECs的距离更远（最近邻距离：31.2 *vs* 24.7 μm，*P*=0.038），且CAFs 30 μm内TAECs的数量更少（接近度：1.1 *vs* 3.6，*P*=0.038）。单因素*Cox*回归显示，低TAECs密度与接受NAIC后MPR有关（OR=36.00, 95%CI: 2.68-1486.88, *P*=0.019）。ROC曲线进一步证实，基线CAFs-TAECs最近邻距离与接近度（30 μm）预测肺鳞状细胞癌NAIC MPR的曲线下面积（area under the curve, AUC）、敏感度和特异度均分别为0.893、0.857和1.000。

**结论:**

CAFs与TAECs的空间距离更远、肺鳞状细胞癌接受NAIC后更易MPR，这可能与CAFs与TAECs的相互作用降低、肿瘤相关血管生成减少有关。

## Abstract (en)

**Background and objective:**

Neoadjuvant therapeutic strategies play a pivotal role in the comprehensive treatment of non-small cell lung cancer (NSCLC). However, lung squamous cell carcinoma (SCC) generally exhibits a more favorable response to neoadjuvant therapy compared with lung adenocarcinoma (ADC). The aim of this study is to elucidate how baseline cancer-associated fibroblasts (CAFs) and tumor-associated endothelial cells (TAECs) influence the differential therapeutic outcomes of neoadjuvant treatment in SCC versus ADC.

**Methods:**

We retrospectively collected pretreatment biopsy samples from 104 patients with stage II-III NSCLC who underwent neoadjuvant chemotherapy (NAC) or neoadjuvant chemoimmunotherapy (NAIC) at Shandong Cancer Hospital between January 1, 2018 and December 31, 2023. Tissue microarrays were constructed using an automated arrayer, and multiplex immunofluorescence staining (α-SMA/CD31/CK/DAPI) was performed to identify CAFs (α-SMA^+^/CK^-^) and TAECs (CD31^+^/CK^-^). Quantitative analyses included CAFs and TAECs densities, the nearest neighbor distance (NND) between CAFs and TAECs, and their spatial proximity (30 μm). Differences in major pathological response (MPR) between groups, defined as residual viable tumor cells ≤10% in resected specimens after neoadjuvant therapy, were assessed using the χ² test. The *Mann-Whitney U* test was applied to analyze intergroup differences in quantitative indicators, and receiver operating characteristic (ROC) curve analysis was conducted to evaluate the predictive performance of immune-related markers for MPR in the NAIC cohort.

**Results:**

Among the 104 NSCLC patients who received neoadjuvant therapy, 35 underwent NAIC and 69 received NAC. Overall, patients with SCC were more likely to achieve MPR compared with those with ADC (50.0% *vs* 22.4%, *P*=0.006). This trend persisted in the NAIC subgroup (72.7% *vs* 30.8%, *P*=0.038), whereas no significant difference in MPR rates was observed between SCC and ADC in the NAC subgroup. At baseline, prior to NAIC or NAC, programmed cell death ligand 1 (PD-L1)/programmed cell death 1 (PD-1) expression, CAFs and TAECs densities, CAFs-TAECs NND, and CAFs-TAECs proximity (30 μm) showed no significant differences between SCC and ADC. In patients with SCC receiving NAIC, baseline PD-L1/PD-1 expression, CAFs density, and TAECs density showed not significant differences between MPR and NMPR groups. However, the CAFs-TAECs distance was significantly greater in the MPR group (NND: 31.2 *vs* 24.7 μm, *P*=0.038), and the number of TAECs within 30 μm of CAFs was significantly lower (proximity: 1.1 *vs* 3.6, *P*=0.038). Univariate *Cox* regression analysis indicated that low TAECs density was associated with MPR following NAIC (OR=36.00, 95%CI: 2.68-1486.88, *P*=0.019). Furthermore, ROC analysis demonstrated that baseline CAFs-TAECs NND and proximity (30 μm) exhibited strong predictive performance for MPR in SCC patients treated with NAIC, with an area under the curve (AUC) of 0.893, sensitivity of 0.857, and specificity of 1.000.

**Conclusion:**

CAFs are more spatially distant from TAECs and more prone to MPR after NAIC in SCC, which may be related to the reduced interaction of CAFs with TAECs and reduced tumor-associated angiogenesis.

非小细胞肺癌（non-small cell lung cancer, NSCLC）约占所有肺癌的82%，其中约40%的患者可进行根治性手术，但单纯手术复发及转移率高，术后5年生存率仅为19%-52%^[1]^。放疗、化疗及联合放化疗的治疗效果并不理想，总生存率不超过50%^[2]^。新辅助治疗策略在局部晚期肺癌的综合治疗中具有重要潜力，为改善患者预后提供了新方向。研究^[3,4]^已证实新辅助治疗的围手术期安全性和耐受性良好。新辅助化疗（neoadjuvant chemotherapy, NAC）具有更早治疗微小转移性病灶，以及使患者更容易完成计划化疗疗程等优点^[5]^。相较于NAC，新辅助化免（neoadjuvant chemoimmunotherapy, NAIC）可以显著提高NSCLC患者的完全病理缓解（pathological complete response, pCR）、主要病理缓解（major pathological response, MPR）和无事件生存期^[6,7]^。然而研究^[8,9]^表明，肺鳞状细胞癌患者对新辅助治疗的疗效普遍优于肺腺癌，尤其在达到MPR的患者中，肺鳞状细胞癌患者比例显著更高。

肿瘤微环境（tumor microenvironment, TME）与NSCLC的发生发展密切相关^[10]^。癌症相关成纤维细胞（cancer-associated fibroblasts, CAFs）是一种存在于多种肿瘤TME中的基质细胞，通过分泌生长因子和其他信号分子来促进肿瘤存活和生长并刺激肿瘤细胞分裂和生长^[11]^。肿瘤相关血管内皮细胞（tumor-associated endothelial cells, TAECs）在血管生成中起着关键作用，而血管为肿瘤细胞提供代谢支持，并作为它们发生血行转移的关键门户，因此TAECs对肿瘤的生长与侵袭至关重要^[12]^，已有研究^[13,14]^证明，CAFs标志物和高肿瘤微血管密度（microvascular density, MVD）与NSCLC较差生存期相关。在肺腺癌中，CAFs的丰度与MVD呈正相关，并且CAFs可以通过促进血管生成从而促进其进展^[15]^。评估CAFs与TAECs的密度及空间距离，可为接受新辅助治疗的鳞状细胞癌与腺癌患者提供更为准确的疗效信息。

多色免疫荧光技术允许在单细胞分辨率下同时检测多种蛋白，是分析细胞密度及空间距离的可靠工具^[16-18]^。应用多色免疫荧光技术对CAFs和TAECs的密度与空间距离进行定量分析，本研究旨在探究接受新辅助治疗的鳞状细胞癌与腺癌患者出现疗效差异的原因，以指导个体化治疗。

## 1 资料与方法

### 1.1 患者及样本来源

回顾性纳入104例于2018年1月1日至2023年12月31日在山东省肿瘤医院接受NAIC或NAC治疗的NSCLC患者，收集治疗前福尔马林固定、石蜡包埋的原发肿瘤标本及其临床病理资料。患者纳入标准：（1）美国癌症联合委员会第八版病理分期为IIA-IIIB期的原发性NSCLC患者；（2）在本院接受根治性肺叶切除手术；（3）随访资料和病理标本可获得。排除标准：（1）同时存在其他恶性肿瘤；（2）缺乏完整的临床资料（相关临床资料从病历中获取）；（3）失访；（4）标本不完整。本研究经山东省肿瘤医院伦理审查委员会批准（审批号：SDTHEC2023003055）。

### 1.2 多色免疫荧光染色与免疫组织化学染色

多色免疫荧光染色指标为程序性细胞死亡配体1（programmed cell death ligand 1, PD-L1）、程序性细胞死亡受体1（programmed cell death 1, PD-1）、α-SMA、CD31、CK和DAPI。染色步骤如下：（1）脱蜡水化：切片置于65 ^o^C烤箱中2 h，二甲苯脱蜡后依次通过体积分数为100%、95%和70%的乙醇再水化；（2）抗原修复：柠檬酸缓冲液或乙二胺四乙酸缓冲液（均按说明书稀释）抗原修复15 min；（3）封闭：滴加封闭液，室温PD-L1 30 min、PD-1 30 min、α-SMA 30 min、CD31 30 min、CK 20 min；（4）一抗孵育：滴加一抗溶液[PD-L1（13684S, 1:200）、PD-1（ZM-0381, 1:500）、α-SMA（Abcam, 1:300）、CD31（Abcam, 1:2000）、CK（ZSGB-BIO, 1:1600）]，并在室温孵育1 h；TSBT洗涤3次，5 min/次。（5）二抗孵育：二抗工作液室温孵育20 min；再次用TBST洗脱二抗；（6）PPD与Opal染料：滴加PPD染料[PD-L1（1:200）、α-SMA（1:200）和CK（1:200）]与Opal染料[PD-1（1:150）、CD31（1:150）]，室温孵育10 min；（7）重复步骤（2）至（6）2次；（8）DAPI（Akoya Biosciences, 1:100）复染，室温孵育5 min；（9）封片。

免疫组织化学染色指标为PD-L1。平台为Dako Autostainer Link 48。染色步骤如下：（1）脱蜡水化：切片置于65 ^o^C烤箱中2 h，二甲苯脱蜡后依次通过体积分数为100%、95%和70%的乙醇再水化；（2）抗原修复：柠檬酸缓冲液（即用型）抗原修复20 min；（3）冷却与阻断：自然冷却至室温后过氧化氢阻断剂10 min；（4）一抗孵育：滴加一抗溶液（克隆22C3，即用型），并在室温孵育1 h；TSBT洗涤3次，5 min/次。（5）二抗孵育：二抗工作液室温孵育30 min；再次用TBST洗脱二抗；（6）显色：滴加DAB染料（即用型），室温孵育10 min；（7）复染：苏木精复染10 min；（8）封片。（9）阴性试剂对照：重复步骤（1）至（8）。评分标准采用肿瘤细胞阳性比例分数（tumor proportion score, TPS）。TPS定义为PD-L1染色阳性的存活肿瘤细胞数在所有存活的肿瘤细胞总数所占百分比。判读标准为TPS<1%、TPS 1%-49%、TPS≥50%。

### 1.3 图像扫描分析

采用Akoya Polaris全景组织多光谱成像系统对染色切片的荧光信号进行全景扫描，其内置inFrom软件进行细胞密度、最近邻距离和接近度的定量分析，随后使用R 3.6.3进行数据整合并导出。DAPI用于标记细胞核，CK用于标记上皮细胞。细胞密度计算为每1000个细胞内的特定细胞表征数^[16]^。PD-L1、PD-1表达用细胞密度计算。最近邻距离定义为每个α-SMA^+^细胞与其最近相邻的CD31^+^细胞之间的平均距离^[17]^，单位为μm。接近度定义为以α-SMA^+^细胞为圆心，30 μm半径内至少存在1个CD31^+^细胞的α-SMA^+^总数^[17]^。选择30 μm为半径，是因为其可能是细胞与细胞之间发生直接/间接相互作用的生理学合适距离^[18]^。

### 1.4 疗效评估

客观缓解率（objective response rate, ORR）定义为根据实体瘤疗效评价标准1.1（Response Evaluation Criteria in Solid Tumors 1.1, RECIST 1.1）获得完全缓解或部分缓解的患者比例^[7]^。MPR定义为经新辅助治疗后，术后切除标本瘤床内的残存活肿瘤细胞的百分比≤10%，无论淋巴结内有无活肿瘤细胞残存；NMPR则定义为>10%^[7]^。pCR定义为患者在接受新辅助治疗后，术后切除标本瘤床内和淋巴结内均无残存活肿瘤细胞^[6]^。

### 1.5 统计学分析

采用SPSS 23.0及GraphPad Prism 10.1.1对数据进行统计学描述及分析。χ^2^检验及*Fisher*检验比较分类变量的组间差异。采用*Mann-Whitney U*检验分析连续变量组间差异，数据以中位数和四分位数形式呈现。单因素Cox回归分析接受NAIC治疗后MPR的影响因素。受试者工作特征（receiver operating characteristic, ROC）曲线确定TME特征对MPR的预测效能。检验水准α=0.05（双尾），以*P*<0.05为差异有统计学意义。

## 2 结果

### 2.1 患者临床及病理特征

本研究共纳入104例NSCLC患者，其中肺鳞状细胞癌46例（44.2%），肺腺癌58例（55.8%）。患者群体以<65岁（66.3%）、男性（82.7%）为主，肿瘤位置以周围型（76.0%）为主。肺鳞状细胞癌组与肺腺癌组在年龄、性别、吸烟指数、肿瘤位置、胸膜侵犯、表皮生长因子受体（epidermal growth factor receptor, EGFR）状态、美国东部肿瘤合作组体能状态（Eastern Cooperative Oncology Group performance status, ECOG PS）评分、cN分期、cM分期和pT分期方面均无统计学差异（*P*>0.05）（表1）；在cT分期、pN分期、免疫检查点抑制剂（immune checkpoint inhibitors, ICIs）类型和TPS评分方面有统计学差异（*P*<0.05）（表1）。

**表1:** 肺腺癌与肺鳞状细胞癌患者的基线特征

Characteristics	All	SCC	ADC	P
	(*n*=104)	(*n*=46)	(*n*=58)	
Age				0.994
<65 yr	69 (66.3%)	30 (65.2%)	39 (67.2%)	
≥65 yr	35 (33.7%)	16 (34.8%)	19 (32.8%)	
Gender				0.199
Female	18 (17.3%)	5 (10.9%)	13 (22.4%)	
Male	86 (82.7%)	41 (89.1%)	45 (77.6%)	
Smoking index^a^				0.287
<400	41 (39.4%)	15 (32.6%)	26 (44.8%)	
≥400	63 (60.6%)	31 (67.4%)	32 (55.2%)	
Tumor location				0.838
Central	25 (24.0%)	12 (26.1%)	13 (22.4%)	
Peripheral	79 (76.0%)	34 (73.9%)	45 (77.6%)	
Pleural invasion				0.919
Yes	39 (37.5%)	18 (39.1%)	21 (36.2%)	
No	65 (62.5%)	28 (60.9%)	37 (63.8%)	
EGFR status				0.999
Mutation	0 (0.0%)	0 (0.0%)	0 (0.0%)	
Wild	51 (49.0%)	23 (50.0%)	28 (48.3%)	
Not clear	53 (51.0%)	23 (50.0%)	30 (51.7%)	
ECOG PS				NA
0-1	104 (100.0%)	46 (100.0%)	58 (100.0%)	
cT stage				0.020
cT_1_	10 (9.6%)	2 (4.3%)	8 (13.8%)	
cT_2_	48 (46.2%)	16 (34.8%)	32 (55.2%)	
cT_3_	28 (26.9%)	17 (37.0%)	11 (19.0%)	
cT_4_	18 (17.3%)	11 (23.9%)	7 (12.0%)	
cN stage				0.540
cN_0_	16 (15.4%)	9 (19.6%)	7 (12.1%)	
cN_1_	20 (19.2%)	9 (19.6%)	11 (19.0%)	
cN_2_	60 (57.7%)	26 (56.5%)	34 (58.6%)	
cN_3_	8 (7.7%)	2 (4.3%)	6 (10.3%)	
cM stage				0.558
cM_0_	103 (99.0%)	46 (100.0%)	57 (98.3%)	
cM_x_	1 (1.0%)	0 (0.0%)	1 (1.7%)	
pT stage				0.123
pT_0_	12 (11.5%)	9 (19.6%)	3 (5.2%)	
pT_1_	24 (23.1%)	10 (21.7%)	14 (24.1%)	
pT_2_	42 (40.4%)	16 (34.8%)	26 (44.8%)	
pT_3_	18 (17.3%)	6 (13.0%)	12 (20.7%)	
pT_4_	8 (7.7%)	5 (10.9%)	3 (5.2%)	
pN stage				<0.001
pN_0_	49 (47.1%)	31 (67.4%)	18 (31.0%)	
pN_1_	15 (14.4%)	7 (15.2%)	8 (13.8%)	
pN_2_	37 (35.6%)	8 (17.4%)	29 (50.0%)	
pN_3_	3 (2.9%)	0 (0.0%)	3 (5.2%)	
ICIs type				0.010
Anti-PD-1	32 (30.8%)	19 (41.3%)	13 (22.4%)	
Anti-PD-L1	3 (2.9%)	3 (6.5%)	0 (0.0%)	
Non-anti-PD-1/anti-PD-L1	69 (66.3%)	24 (52.2%)	45 (77.6%)	
TPS				0.031
<1%	1 (0.9%)	1 (2.2%)	0 (0.0%)	
1%-49%	9 (8.7%)	7 (15.2%)	2 (3.4%)	
≥50%	7 (6.7%)	5 (10.9%)	2 (3.4%)	
Unknown	87 (83.7%)	33 (71.7%)	54 (93.2%)	

^a^Smoking index means duration of smoking (years)×number of cigarettes smoked per year (cigarettes); Non-anti-PD-1/anti-PD-L1 means the patients do not received ICIs treatment. SCC: squamous cell carcinoma; ADC: adenocarcinoma; ECOG PS: Eastern Cooperative Oncology Group performance status; EGFR: epidermal growth factor receptor; PD-1: programmed cell death 1; PD-L1: programmed death ligand 1; ICIs: immune checkpoint inhibitors; TPS: tumor proportion score; NA: not applicable.

### 2.2 肺鳞状细胞癌与肺腺癌NAIC/NAC治疗后ORR、MPR及pCR差异

在104例接受新辅助治疗的NSCLC患者中，肺鳞状细胞癌与肺腺癌的ORR无统计学差异。然而，肺鳞状细胞癌的MPR及pCR显著优于肺腺癌，表明肺鳞状细胞癌对新辅助治疗更为敏感（表2）。

**表2:** 不同组织学亚型中NAIC与NAC疗效差异

Index	All (*n*=104)			NAIC (*n*=35)			NAC (*n*=69)		
	SCC (*n*=46)	ADC (*n*=58)	*P*	SCC (*n*=22)	ADC (*n*=13)	*P*	SCC (*n*=24)	ADC (*n*=45)	*P*
ORR			0.301			0.696			0.999
Yes	23 (50.0%)	22 (37.9%)		13 (59.1%)	6 (46.2%)		10 (41.7%)	16 (35.6%)	
No	23 (50.0%)	36 (62.1%)		9 (40.9%)	7 (53.8%)		14 (58.3%)	29 (64.4%)	
MPR			0.006			0.038			0.903
Yes	23 (50.0%)	13 (22.4%)		16 (72.7%)	4 (30.8%)		7 (29.2%)	9 (20.0%)	
No	23 (50.0%)	45 (77.6%)		6 (27.3%)	9 (69.2%)		17 (70.8%)	36 (80.0%)	
pCR			0.025			0.150			0.999
Yes	10 (21.7%)	3 (5.2%)		9 (40.9%)	2 (15.4%)		1 (4.2%)	1 (2.2%)	
No	36 (78.3%)	55 (94.8%)		13 (59.1%)	11 (84.6%)		23 (95.8%)	44 (97.8%)	

ORR: objective response rate; MPR: major pathological response; pCR: pathological complete response; NAIC: neoadjuvant chemoimmunotherapy; NAC: neoadjuvant chemotherapy.

接下来，我们进一步分析了肺鳞状细胞癌和肺腺癌在不同新辅助治疗方式中的疗效差异。在35例接受NAIC的患者中，肺鳞状细胞癌的MPR显著优于肺腺癌（*P*<0.05），而ORR与pCR虽数值上肺鳞状细胞癌更高，但无统计学差异（*P*>0.05）。在69例接受NAC的患者中，肺鳞状细胞癌与肺腺癌的ORR、MPR及pCR均无差异（*P*>0.05）（表2）。

### 2.3 肺鳞状细胞癌与肺腺癌NAIC/NAC治疗前瘤内CAFs及TAECs的差异

为了分析在接受NAIC与NAC的患者中，肺鳞状细胞癌与肺腺癌患者治疗前瘤内CAFs、TAECs的密度、最近邻距离以及接近度差异，我们利用多色免疫荧光技术对14例NAIC和22例NAC患者治疗前活检样本的肿瘤区域进行了α-SMA与CD31表达检测。结果显示，α-SMA主要表达在细胞质，CD31主要表达在细胞膜（图1）。CAFs定义为α-SMA^+^/CK^-^，TAECs定义为CD31^+^/CK^-^（图1）。结果显示，在接受NAIC与NAC的患者中，肺鳞状细胞癌与肺腺癌患者治疗前PD-L1、PD-1表达、CAFs和TAECs的密度、最近邻距离以及接近度均无差异（*P*>0.05）（表3）。

**图1:**
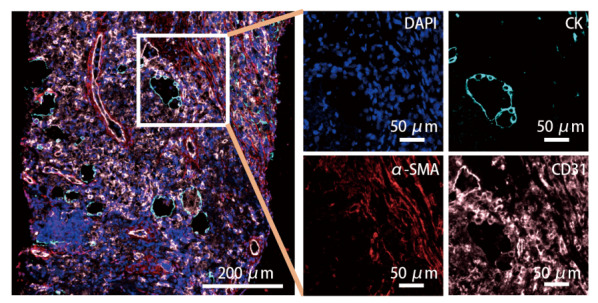
1例接受NAIC治疗的肺鳞状细胞癌患者术前活检标本的多色免疫荧光图像。 DAPI：蓝色；CK：蓝绿色；α-SMA：红色；CD31：粉红色。

**表3:** 基线NAIC与NAC的肺鳞状细胞癌与肺腺癌患者TME特征差异

TME characteristics	NAIC			NAC		
	SCC (*n*=11)	ADC (*n*=3)	*P*	SCC (*n*=7)	ADC (*n*=15)	*P*
PD-L1 expression (cell counts)	110 (30, 150)	30 (15, 125)	0.583	0 (0, 1)	1 (0, 10)	0.336
PD-1 expression (cell counts)	0 (0, 25)	20 (10, 40)	0.559	3 (2, 55)	3 (0, 14)	0.520
CAFs density (cell counts)	588 (370, 631)	437 (397, 450)	0.586	379 (296, 467)	515 (303, 770)	0.275
TAECs density (cell counts)	117 (81, 270)	312 (258, 343)	0.139	95 (59, 228)	151 (43, 234)	0.805
NND (μm)	29.6 (24.7, 31.8)	17.4 (16.7, 18.7)	0.052	28.3 (23.7, 41.1)	33.2 (22.4, 55.3)	0.698
Proximity (30 μm) (cell counts)	2.5 (1.0, 3.6)	4.2 (4.1, 4.5)	0.186	2.5 (1.4, 2.5)	2.5 (0.8, 3.8)	0.597

TME: tumor microenvironment; CAFs: cancer-associated fibroblasts; TAECs: tumor-associated endothelial cells; NND: nearest neighbor distance.

### 2.4 NAIC与NAC治疗前瘤内CAFs及TAECs的差异

整体队列及肺鳞状细胞癌亚组中，NAIC组的PD-L1表达显著高于NAC组（All: 90 *vs* 0, *P*=0.001; SCC: 110 *vs* 0, *P*=0.004），而在肺腺癌亚组中无统计学差异（*P*>0.05）。而PD-1表达、TAECs密度、CAFs密度、最近邻距离及邻近度（30 μm）均无统计学差异（*P*>0.05）（表4）。

**表4:** NAIC与NAC基线TME差异

TME characteristics	All (*n*=36)			SCC (*n*=18)			ADC (*n*=18)		
	NAIC (*n*=14)	NAC (*n*=22)	*P*	NAIC (*n*=11)	NAC (*n*=7)	*P*	NAIC (*n*=3)	NAC (*n*=15)	*P*
PD-L1 expression (cell counts)	90 (30, 165)	0 (0, 8)	0.001	110 (30, 150)	0 (0, 1)	0.004	30 (15, 125)	1 (0, 10)	0.264
PD-1 expression (cell counts)	10 (0, 27)	3 (0, 16)	0.816	0 (0, 25)	3 (2, 55)	0.327	20 (10, 40)	3 (0, 14)	0.469
CAFs density (cell counts)	450 (364, 599)	459 (280, 662)	0.922	588 (370, 631)	379 (296, 466)	0.258	437 (396, 450)	515 (303, 770)	0.515
TAECs density (cell counts)	176 (94, 342)	130 (49,241)	0.408	117 (80, 270)	95 (58, 227)	0.526	312 (258, 342)	151 (43, 233)	0.173
NND (μm)	28.2 (18.0, 31.0)	31.6 (21.7, 47.7)	0.256	29.6 (24.7, 31.8)	28.3 (23.7, 41.1)	0.964	17.4 (16.7, 18.7)	33.2 (22.4, 55.3)	0.051
Proximity (30 μm) (cell counts)	2.6 (1.3, 4.4)	2.5 (0.8, 3.5)	0.455	2.5 (1.0, 3.6)	2.5 (1.4, 2.5)	0.821	4.2 (4.1, 4.5)	2.5 (0.8, 3.8)	0.110

### 2.5 CAFs及TAECs对肺鳞状细胞癌及肺腺癌NAC/NAIC治疗后MPR的影响

在接受NAIC治疗的肺鳞状细胞癌患者中，与NMPR患者相比，MPR患者治疗前PD-L1、PD-1表达（PD-L1: 120 *vs* 70, *P*=0.343; PD-1: 20 *vs* 0, *P*=0.215）、CAFs及TAECs的密度评分无统计学差异（CAFs: 588 *vs* 525, *P*=0.705; TAECs: 90 *vs* 270, *P*=0.089），CAFs与TAECs的距离更远（31.2 *vs* 24.7 μm, *P*=0.038），且CAFs 30 μm内TAECs的数量更低（1.1 *vs* 3.6, *P*=0.038）（图2，表5）。由于仅有3例肺腺癌接受NAIC治疗，因此未行接受NAIC治疗的肺腺癌患者的MPR与NMPR的组间差异分析。

**图2:**
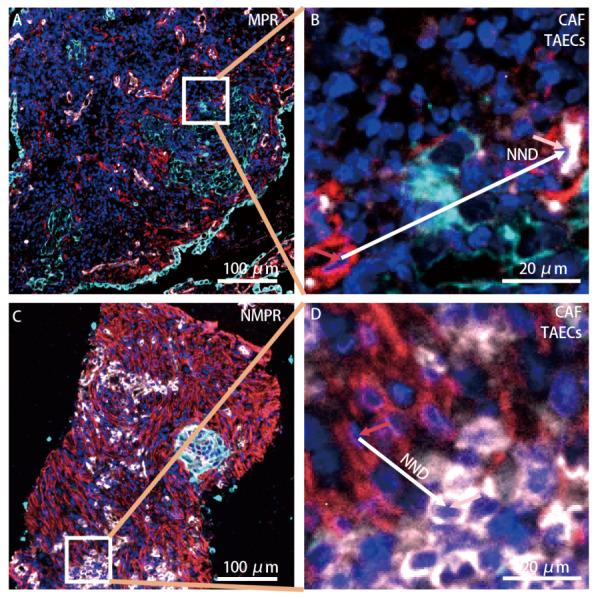
NAIC治疗前肺鳞状细胞癌MPR（A）与NMPR（C）者CAFs- TAECs最近邻距离示意图。肺鳞状细胞癌MPR者（B）CAFs- TAECs最近邻距离明显长于NMPR者（D）。

**表5:** 基线肺鳞状细胞癌与肺腺癌的MPR与NMPR患者TME特征差异

TME characteristics	NAIC of SCC			NAC of ADC		
	MPR (*n*=7)	NMPR (*n*=4)	*P*	MPR (*n*=3)	NMPR (*n*=12)	*P*
PD-L1 expression (cell counts)	120 (50, 200)	70 (25, 112)	0.343	0 (0, 0)	2 (0, 14)	0.068
PD-1 expression (cell counts)	20 (0, 40)	0 (0, 5)	0.215	0 (0, 1)	4 (0, 20)	0.106
CAFs density (cell counts)	588 (376, 598)	525 (379, 687)	0.705	258 (185, 363)	630 (406, 796)	0.083
TAECs density (cell counts)	90 (53, 141)	270 (167, 429)	0.089	151 (87, 280)	154 (46, 225)	0.885
NND (μm)	31.2 (29.9, 70.2)	24.7 (20.1, 28.1)	0.038	21.0 (18.8, 52.8)	34.3 (27.8, 50.5)	0.564
Proximity (30 μm) (cell counts)	1.1 (0.6, 2.1)	3.6 (2.6, 5.2)	0.038	3.4 (2.0, 4.6)	1.8 (0.8, 3.7)	0.564

在接受NAC治疗的肺腺癌患者中，与NMPR患者相比，MPR患者治疗前PD-L1、PD-1表达（PD-L1: 0 *vs* 2, *P*=0.068; PD-1: 0 *vs* 4, *P*=0.106）、CAFs及TAECs的密度（CAFs: 258 *vs* 630, *P*=0.083; TAECs: 151 *vs* 154, *P*=0.885）、距离（21.0 *vs* 34.3 μm, *P*=0.564）和接近度（3.4 *vs* 1.8, *P*=0.564）均无统计学差异（表5）。由于仅有7例肺鳞状细胞癌患者接受NAC治疗，未行接受NAC治疗的肺鳞状细胞癌患者的MPR与NMPR的组间差异分析。

### 2.6 低TAECs密度与接受NAIC后MPR有关

基于年龄、性别、吸烟指数、组织学类型、肿瘤位置等临床因素和TAECs、CAFs的密度、最近邻距离、接近度（30 μm）以及PD-L1、PD-1表达等进行单因素Cox回归分析（表6）。单因素分析结果显示低TAECs密度与接受NAIC后MPR有关（OR=36.00, 95%CI: 2.68-1486.88, *P*=0.019），而其他临床特征与TME特征均与接受NAIC后MPR无关（*P*>0.05）（表6）。

**表6:** NAIC MPR与NMPR患者影响因素分析

Variables	OR (95%CI)	P
Age (>65 yr *vs* ≤65 yr)	1.78 (0.22-16.40)	0.594
Gender (Male *vs* Female)	2.40 (0.18-61.34)	0.522
Smoking index^a^ (≥400 *vs* <400)	1.00 (0.11-8.76)	0.999
Histology subtype (SCC *vs* ADC)	**NA**	0.996
cT stage (cT_3_ *vs* cT_2_+cT_4_）	5.00 (0.41-131.10)	0.239
cN stage (cN_1_ *vs* cN_2_+cN_3_）	2.00 (0.09-69.06)	0.661
pT stage (pT_1_ *vs* pT_2_+pT_3_）	**NA**	0.997
pN stage (pN_1_ *vs* pN_2_+pN_3_）	**NA**	0.998
Tumor location (Peripheral *vs* Central)	**NA**	0.995
Visceral pleural invasion (Yes *vs* No)	**NA**	0.995
ICIs type (Anti-PD-L1 *vs* Anti-PD-1)	0.42 (0.02-5.68)	0.522
PD-L1 expression (Low *vs* High)	0.22 (0.01-2.48)	0.256
PD-1 expression (Low *vs* High)	0.22 (0.01-2.48)	0.256
CAFs density (Low *vs* High)	0.30 (0.03-2.58)	0.288
TAECs density (Low *vs* High)	36.00 (2.68-1486.88)	0.019
NND (Near *vs* Far)	**NA**	0.998
Proximity (30 μm) (Low *vs* High)	**NA**	0.998

^a^Smoking index=duration of smoking (years)×number of cigarettes smoked per year (cigarettes).

### 2.7 CAFs与TAECs的距离及接近度决定肺鳞状细胞癌NAIC后MPR

为进一步验证基线TME特征对NAIC治疗肺鳞状细胞癌患者病理缓解的预测价值，我们对MPR与NMPR组间存在差异显著的特征进行了ROC曲线分析。结果显示，CAFs与TAECs的距离及接近度（30 μm）均表现出优异的预测效能，ROC曲线下面积（area under the curve, AUC）均达0.893。在最佳截断值下，两者灵敏度为0.857，特异度为1.000。这表明基线状态下CAFs-TAECs的距离及接近度（30 μm）可作为NAIC治疗后肺鳞状细胞癌患者达到MPR的独立预测因子（图3）。

**图3:**
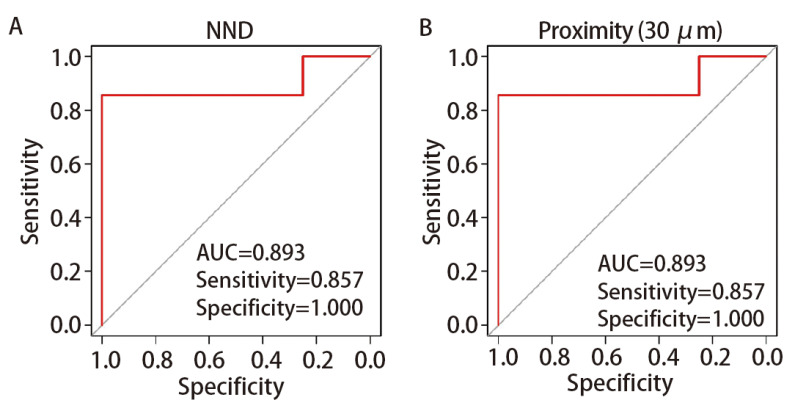
CAFs与TAECs的最近邻距离（A）与接近度(30 μm)（B）预测肺鳞状细胞癌NAIC治疗后MPR。

## 3 讨论

本研究探讨了II-III期肺鳞状细胞癌与腺癌患者对不同新辅助治疗方式的疗效差异及基线CAFs与TAECs对其治疗疗效的影响，以指导个体化治疗。肺鳞状细胞癌对新辅助治疗的疗效较肺腺癌更好，尤其是在接受NAIC治疗的情况下。在接受NAIC治疗的肺鳞状细胞癌患者中，MPR较NMPR患者基线的CAFs-TAECs距离更远，并可预测MPR。而在接受NAC治疗的肺腺癌患者中，其均无显著差异。同时，低TAECs密度与接受NAIC后MPR有关。

已有研究证明，肺鳞状细胞癌患者接受新辅助治疗的疗效普遍比肺腺癌更好。在接受NAC治疗的患者中，肺鳞状细胞癌患者的MPR优于肺腺癌^[8,20]^，而在接受NAIC治疗并出现MPR的患者中，有研究观察到了更多的肺鳞状细胞癌患者^[9]^。我们的研究中接受NAC治疗的肺腺癌患者并没有观察到疗效差异，而接受NAIC治疗的患者却观察到了相同的现象。这又进一步验证了不同病理学亚型的NSCLC对NAIC治疗反应的特异性。

既往研究发现，CAFs和TAECs作为肿瘤支持细胞，通常与患者不良预后相关^[13,14]^，而新辅助治疗联合抗血管生成药物可提升疗效^[21]^。本研究中，我们虽未观察到MPR与NMPR患者间CAFs密度的显著差异，但发现接受NAIC治疗的肺鳞状细胞癌患者中，NMPR患者的TAECs密度呈现高于MPR患者的趋势（尽管无显著统计学差异）。这一发现与既往研究^[13,14]^相符。后续研究需扩大样本，以进一步验证高TAECs密度对NSCLC患者生存期的潜在不利影响。

利用多色免疫荧光，多项研究证实TME特征是预测NSCLC NAIC疗效的关键。Bai等^[22]^研究表明，肺门淋巴结树突状细胞密度与TME中CD8^+^ T细胞密度同步升高，可显著提升NSCLC患者的pCR和无病生存期。Yang等^[23]^发现，基线TME中CD4^+^与CD8^+^ T细胞的邻近距离缩短能显著增强PD-L1低表达NSCLC患者NAIC的疗效，且该效应与MVD呈正相关。然而，既往研究^[24]^多聚焦于TME特征对患者生存及预后的影响，而很少有关联其与不同新辅助治疗下鳞状细胞癌与腺癌患者疗效差异的研究。此外，多数研究^[13,14,24]^仅关注CAFs与TAECs的密度，却忽视了二者空间距离对生存、预后及治疗反应的潜在作用。因此，本研究不仅探究了CAFs与TAECs密度对新辅助治疗疗效的影响，更特别关注了二者间空间距离对疗效的作用。

在接受NAIC治疗后MPR的肺鳞状细胞癌患者中，CAFs与TAECs的最近邻距离较NMPR患者更长，而接近度则相对更弱，表明在MPR患者中，两者的空间距离相对更远。肺腺癌相关研究^[14]^发现，CAFs表达的四半LIM结构域蛋白2（four and a half LIM domains protein 2, FHL2），作为一种蛋白质相互作用介导因子，可促进血管生成。此外，基质FHL2高表达与升高的MVD显著相关。因此，我们推测，在接受NAIC治疗的肺鳞状细胞癌患者中，MPR患者疗效优于NMPR患者的原因可能在于：MPR患者基线状态下CAFs与TAECs的相互作用较弱。这种较弱的相互作用可能导致CAFs表达的FHL2的促血管生成效应在MPR患者中相应减弱，最终使得MPR患者的MVD维持在较低水平。低MVD导致疗效较好的原因主要可能有以下几个方面，一是血管内皮生长因子会导致肿瘤组织微血管渗透压与组织间液压升高，从而导致药物递送效率低下，而低MVD状态通常伴随血管内皮生长因子水平下调，从而提升药物递送效率^[25]^。二是MVD已被证明与肿瘤内坏死有关，引发局部缺氧与坏死灶形成，促进免疫抑制微环境^[26]^。而低MVD使肿瘤缺氧程度显著缓解。但此推测需要进一步通过体内外实验证明。

本研究揭示了II-III期肺鳞状细胞癌患者中CAFs与TAECs的密度及空间距离对NAIC疗效的预测价值，其中CAFs与TAECs空间距离更远、接受NAIC治疗的肺鳞状细胞癌患者更易出现MPR。这可能有助于指导不同病理学亚型的NSCLC患者的个性化治疗策略。

本研究存在一些局限性。首先，受限于部分患者治疗前标本缺失等客观因素，回顾性设计固有的选择偏倚难以避免。其次，样本量有限，尤其在具有配对活检样本并接受NAIC的肺鳞状细胞癌以及接受NAC的肺腺癌患者亚群中，这可能限制了结果的普遍性和统计效力。因此，未来研究需在更大规模的前瞻性队列中验证这些发现，并深入探索其潜在生物学机制。

## References

[1] Siegel RL, Miller KD, Wagle NS, et al. Cancer statistics, 2023. CA Cancer J Clin, 2023, 73(1): 17-48. doi: 10.3322/caac.21763 36633525

[2] Zhang YS, Han Y, Che NY, et al. Preliminary study on the efficacy of neoadjuvant immunotherapy combined with chemotherapy in locally advanced non-small cell lung cancer. Zhongguo Yikan, 2022, 57(9): 1016-1020.

[3] Huang Z, Wu Z, Qin Y, et al. Perioperative safety and feasibility outcomes of stage IIIA-N2 non-small cell lung cancer following neoadjuvant immunotherapy or neoadjuvant chemotherapy: a retrospective study. Ann Transl Med, 2021, 9(8): 685. doi: 10.21037/atm-21-1141 33987383 PMC8106052

[4] Cascone T, William WN, Weissferdt A, et al. Neoadjuvant nivolumab or nivolumab plus ipilimumab in operable non-small cell lung cancer: the phase 2 randomized NEOSTAR trial. Nat Med, 2021, 27(3): 504-514. doi: 10.1038/s41591-020-01224-2 33603241 PMC8818318

[5] Miller M, Hanna N. Advances in systemic therapy for non-small cell lung cancer. BMJ, 2021, 375: n2363. doi: 10.1136/bmj.n2363 34753715

[6] Forde PM, Spicer J, Lu S, et al. Neoadjuvant nivolumab plus chemotherapy in resectable lung cancer. N Engl J Med, 2022, 386(21): 1973-1985. doi: 10.1056/NEJMoa2202170 35403841 PMC9844511

[7] Lei J, Zhao J, Gong L, et al. Neoadjuvant camrelizumab plus platinum-based chemotherapy *vs* chemotherapy alone for chinese patients with resectable stage IIIA or IIIB (T3N2) non-small cell lung cancer: the TD-FOREKNOW randomized clinical trial. JAMA Oncol, 2023, 9(10): 1348-1355. doi: 10.1001/jamaoncol.2023.2751 37535377 PMC10401395

[8] Miyata R, Aoki M, Morizono S, et al. Adjuvant chemotherapy in addition to neoadjuvant chemotherapy for locally advanced non-small cell lung cancer. In Vivo, 2024, 38(5): 2515-2522. doi: 10.21873/invivo.13723 39187348 PMC11363782

[9] Wu J, Hou L, E H, et al. Real-world clinical outcomes of neoadjuvant immunotherapy combined with chemotherapy in resectable non-small cell lung cancer. Lung Cancer, 2022, 165: 115-123. doi: 10.1016/j.lungcan.2022.01.019 35123154

[10] Mansouri S, Heylmann D, Stiewe T, et al. Cancer genome and tumor microenvironment: reciprocal crosstalk shapes lung cancer plasticity. Elife, 2022, 11: e79895. doi: 10.7554/eLife.79895 PMC9457687 36074553

[11] Baschieri F, Illand A, Barbazan J, et al. Fibroblasts generate topographical cues that steer cancer cell migration. Sci Adv, 2023, 9(33): eade2120. doi: 10.1126/sciadv.ade2120 PMC10431708 37585527

[12] Bussolati B, Deambrosis I, Russo S, et al. Altered angiogenesis and survival in human tumor-derived endothelial cells. FASEB J, 2003, 17(9): 1159-1161. doi: 10.1096/fj.02-0557fje 12709414

[13] Li K, Wang R, Liu GW, et al. Refining the optimal CAF cluster marker for predicting TME-dependent survival expectancy and treatment benefits in NSCLC patients. Sci Rep, 2024, 14(1): 16766. doi: 10.1038/s41598-024-55375-0 39034310 PMC11271481

[14] Fang L, He Y, Liu Y, et al. Adjustment of microvessel area by stromal area to improve survival prediction in non-small cell lung cancer. J Cancer, 2019, 10(15): 3397. doi: 10.7150/jca.31231 31293643 PMC6603421

[15] Kanzaki R, Reid S, Bolivar P, et al. FHL 2 expression by cancer‐associated fibroblasts promotes metastasis and angiogenesis in lung adenocarcinoma. Int J Cancer, 2025, 156(2): 431-446. doi: 10.1002/ijc.35174 39244734 PMC11578086

[16] Jia K, Chen Y, Sun Y, et al. Multiplex immunohistochemistry defines the tumor immune microenvironment and immunotherapeutic outcome in CLDN18.2-positive gastric cancer. BMC Med, 2022, 20(1): 223. doi: 10.1186/s12916-022-02421-1 35811317 PMC9272556

[17] Mi H, Ho W J, Yarchoan M, et al. Multi-scale spatial analysis of the tumor microenvironment reveals features of cabozantinib and nivolumab efficacy in hepatocellular carcinoma. Front Immunol, 2022, 13: 892250. doi: 10.3389/fimmu.2022.892250 35634309 PMC9136005

[18] Väyrynen SA, Zhang J, Yuan C, et al. Composition, spatial characteristics, and prognostic significance of myeloid cell infiltration in pancreatic cancer. Clin Cancer Res, 2021, 27(4): 1069-1081. doi: 10.1158/1078-0432.CCR-20-3141 33262135 PMC8345232

[19] Zheng X, Weigert A, Reu S, et al. Spatial density and distribution of tumor-associated macrophages predict survival in non-small cell lung carcinoma. Cancer Res, 2020, 80(20): 4414-4425. doi: 10.1158/0008-5472.CAN-20-0069 32699134

[20] Yang G, Cai S, Hu M, et al. Spatial features of specific CD103^+^CD8^+^tissue-resident memory T cell subsets define the prognosis in patients with non-small cell lung cancer. J Transl Med, 2024, 22(1): 27. doi: 10.1186/s12967-023-04839-4 38183111 PMC10770937

[21] Qiao T, Zhao J, Xin X, et al. Combined pembrolizumab and bevacizumab therapy effectively inhibits non-small-cell lung cancer growth and prevents postoperative recurrence and metastasis in humanized mouse model. Cancer Immunol Immunother, 2023, 72(5): 1169-1181. doi: 10.1007/s00262-022-03318-x 36357599 PMC10110651

[22] Bai Z, Cheng X, Ma T, et al. CD8^+^ T cells infiltrating into tumors were controlled by immune status of pulmonary lymph nodes and correlated with non-small cell lung cancer (NSCLC) patients’ prognosis treated with chemoimmunotherapy. Lung Cancer, 2024, 197: 107991. doi: 10.1016/j.lungcan.2024.107991 39454350

[23] Yang L, Geng J, Yang H, et al. Closer proximity of pre-treatment CD4^+^ T cells to CD8^+^ T cells favor response to neoadjuvant immunotherapy in patients with PD-L 1 low-expressing non-small cell lung cancer. Transl Lung Cancer Res, 2025, 14(3): 810-823. doi: 10.21037/tlcr-24-886 40248717 PMC12000951

[24] Schulze AB, Schmidt LH, Heitkötter B, et al. Prognostic impact of CD34 and SMA in cancer-associated fibroblasts in stage I-III NSCLC. Thorac Cancer, 2020, 11(1): 120-129. doi: 10.1111/1759-7714.13248 31760702 PMC6938745

[25] Yin J, Dong F, An J, et al. Pattern recognition of microcirculation with super-resolution ultrasound imaging provides markers for early tumor response to anti-angiogenic therapy. Theranostics, 2024, 14(3): 1312-1324. doi: 10.7150/thno.89306 38323316 PMC10845201

[26] Qiu Z, Wu J, Pang G, et al. CD 34 evaluation of microvasculature in lung adenocarcinoma and its microvascular density predicts postoperative tumor recurrence. Oncol Res, 2025, 31: 1611985. doi: 10.3389/pore.2025.1611985 PMC11788009 39906069

